# Letter to the Editor concerning the article “Application of red light phototherapy in the treatment of radioactive dermatitis in patients with head and neck cancer”

**DOI:** 10.1186/s12957-019-1603-y

**Published:** 2019-03-23

**Authors:** Jolien Robijns, Sandrine Censabella, Stefan Claes, Luc Pannekoeke, Lore Bussé, Dora Colson, Iris Kaminski, Victoria Broux, Joy Lodewijckx, Sofie Puts, Paul Bulens, Annelies Maes, Leen Noé, Marc Brosens, An Timmermans, Ivo Lambrichts, Veerle Somers, Jeroen Mebis

**Affiliations:** 1 Faculty of Medicine and Life Sciences, Hasselt University, Martelarenlaan 42, 3500 Hasselt, Belgium; 2 Department of Medical Oncology, Jessa Hospital, Stadsomvaart 11, Hasselt, Belgium; 3 Limburg Oncology Centre, Jessa Hospital, Stadsomvaart 11, Hasselt, Belgium; 4 Department of Dermatology, Jessa Hospital, Stadsomvaart 11, Hasselt, Belgium

**Keywords:** Photobiomodulation, Acute radiodermatitis, Head and neck cancer, Radiotherapy, Red light

## Abstract

The aim of this Letter to the Editor was to report some methodological shortcomings in the recently published article “Application of red light phototherapy in the treatment of radioactive dermatitis in patients with head and neck cancer” by Zhang et al. There are some issues regarding the incomplete photobiomodulation (PBM) parameters, the chosen outcome measures, and some missing reference articles. In conclusion, the results of this study should be interpreted with caution and further research is necessary.

Dear editor,

We read with great interest the article entitled “Application of red light phototherapy in the treatment of radioactive dermatitis in patients with head and neck cancer” authored by Zhang et al. [1]. The article was published in the *World Journal of Surgical Oncology* in November 2018.

The aim of this study was “to investigate the effect of red light phototherapy (RLPT) on radioactive dermatitis (RD) caused by radiotherapy in patients with head and neck cancer (HNC)” [1]. The authors of the study concluded that RLPT could accelerate wound healing and improve patients’ quality of life [1]. Although the results are interesting, some methodological issues should be considered.

Our research group, under supervision of Prof. Dr. Mebis Jeroen, has already built up some extensive scientific evidence that photobiomodulation therapy (PBMT) is an effective preventive and therapeutic method for acute RD in cancer patients [2–5]. In a recent, randomized, placebo-controlled, clinical trial (RCT; TRANSDERMIS trial), we were able to demonstrate that PBMT can effectively reduce the severity of acute RD in breast cancer patients, both by subjective and objective outcome measures. Moreover, the quality of life of the patients undergoing PBMT was significantly better in comparison with the control group [3, 4]. Currently, we are still performing a RCT investigating the effect of PBMT in HNC patients undergoing radiotherapy (ClinicalTrials.gov Identifier: NCT02738268).

The use of PBMT in the management of acute RD is growing steadily. In order to improve the current manuscript by Zhang et al., we would like to make some suggestions.

1. The preferred name for RLPT is “photobiomodulation therapy (PBMT).” This was determined at the 2014 joint North American Association for Laser Therapy [6] and World Association for Laser Therapy (WALT) conference. PBM is defined as follows: “The therapeutic use of light [e.g., visible, near infrared (NIR), infrared (IR)] absorbed by endogenous chromophores, triggering non-thermal, non-cytotoxic, biological reactions through photochemical or photophysical events, leading to physiological changes” [6].
2. Irradiation and treatment parameters are crucial for a successful PBM treatment. The paper from Zhang et al. did not publish any of these important parameters. As such, it is difficult to know how to interpret the results reported in this manuscript. Therefore, we suggest the authors to include a complete list of PBM parameters as recommended by Jenkins et al. [7].
3. In order to overcome the placebo effect, they should have included a sham treatment in the control group.
4. Concerning the outcome measures, the authors did not mention on which time point they compared the degree of acute RD between the two study groups and if this evaluation was performed in a blinded manner.
5. In their conclusion, they state that RLPT improved the patient’s quality of life. However, they did not evaluate the quality of life of the patients during the trial by using a specific questionnaire. As such, they cannot state that the patient’s quality of life improved due to RLPT, because they have no detailed data [8].
6. Moreover, the authors did not refer to other clinical trials that investigated the use of PBMT in the management of acute RD previous to their study. We suggest that the authors should add these references to their paper [2–4, 9–12].

In conclusion, the study by Zhang et al. demonstrated some disadvantages in their study design and outcome assessment, which raised some questions concerning the value of their conclusion. However, as researchers in the field of PBMT and supportive cancer care, we greatly support all the clinical trials concerning the use of PBM.

## Abbreviations

HNC: Head and neck cancer
IR: Infrared
NAALT: North American Association for Laser Therapy
NIR: Near infrared
PBM: Photobiomodulation therapy
RCT: Randomized, controlled trial
RD: Radioactive dermatitis/radiodermatitis
RLPT: Red light phototherapy
WALT: World Association for Laser Therapy
